# Supplementary material for: Ethics appraisal procedure in 79,670 Marie Skłodowska-Curie proposals from the entire European HORIZON 2020 research and innovation program (2014–2020): A retrospective analysis
Source: PLoS One. 2021 Nov 4;16(11):e0259582. doi: 10.1371/journal.pone.0259582 (PMC8568105; doi:10.1371/journal.pone.0259582)
Supplement: S1 File — (DOCX) [file pone.0259582.s001.docx]

**Supplementary file 1. Frequencies and percentages of all ethics issues categories reported by the applicants in self-assessment table of all submitted proposals, divided per MSCA actions from the entire European HORIZON 2020 research and innovation program (2014 - 2020)**

| **Ethics issues self-declared in COFUND proposals, by category** | **2014** | | **2015** | | **2016** | | **2017** | | **2018** | | **2019** | | **2020** | |
| --- | --- | --- | --- | --- | --- | --- | --- | --- | --- | --- | --- | --- | --- | --- |
|  | % | # | % | # | % | # | % | # | % | # | % | # | % | # |
| PROTECTION OF PERSONAL DATA | 18,02% | 20 | 21,15% | 33 | 21,69% | 36 | 19,53% | 25 | 22,56% | 30 | 22,48% | 29 | 25,00% | 49 |
| NON-EU COUNTRIES | 18,92% | 21 | 17,31% | 27 | 12,65% | 21 | 7,03% | 9 | 8,27% | 11 | 10,08% | 13 | 7,65% | 15 |
| ENVIRONMENT PROTECTION QUESTION | 7,21% | 8 | 10,26% | 16 | 9,64% | 16 | 14,06% | 18 | 9,02% | 12 | 8,53% | 11 | 9,18% | 18 |
| OTHER ETHICS ISSUES | 4,50% | 5 | 3,21% | 5 | 3,01% | 5 | 4,69% | 6 | 4,51% | 6 | 3,10% | 4 | 5,10% | 10 |
| DUAL USE | 1,80% | 2 | 1,92% | 3 | 1,20% | 2 | 1,56% | 2 | 1,50% | 2 | 0,78% | 1 | 1,02% | 2 |
| HUMANS | 14,41% | 16 | 12,82% | 20 | 18,67% | 31 | 17,19% | 22 | 15,79% | 21 | 15,50% | 20 | 19,90% | 39 |
| HUMAN CELLS / TISSUES | 12,61% | 14 | 12,82% | 20 | 10,84% | 18 | 13,28% | 17 | 15,04% | 20 | 16,28% | 21 | 14,29% | 28 |
| ANIMALS | 17,12% | 19 | 16,03% | 25 | 16,27% | 27 | 14,84% | 19 | 16,54% | 22 | 18,60% | 24 | 14,29% | 28 |
| MISUSE | 1,80% | 2 | 1,28% | 2 | 1,81% | 3 | 0,78% | 1 | 1,50% | 2 | 0,78% | 1 | 0,51% | 1 |
| HUMAN EMBRYOS/FOETUS | 3,60% | 4 | 3,21% | 5 | 3,01% | 5 | 6,25% | 8 | 4,51% | 6 | 3,10% | 4 | 2,55% | 5 |
| CIVIL APPLICATIONS | 0,00% | 0 | 0,00% | 0 | 1,20% | 2 | 0,78% | 1 | 0,75% | 1 | 0,78% | 1 | 0,51% | 1 |

| **Ethics issues self-declared in IF proposals, by category** | **2014** | | **2015** | | **2016** | | **2017** | | **2018** | | **2019** | | **2020** | |
| --- | --- | --- | --- | --- | --- | --- | --- | --- | --- | --- | --- | --- | --- | --- |
|  | % | # | % | # | % | # | % | # | % | # | % | # | % | # |
| PROTECTION OF PERSONAL DATA | 15,43% | 898 | 17,41% | 987 | 18,96% | 1139 | 19,34% | 1279 | 20,21% | 1535 | 20,86% | 1606 | 21,66% | 1987 |
| NON-EU COUNTRIES | 24,99% | 1454 | 13,67% | 775 | 12,42% | 746 | 12,70% | 840 | 11,64% | 884 | 11,98% | 922 | 13,04% | 1196 |
| ENVIRONMENT PROTECTION QUESTION | 7,65% | 445 | 8,98% | 509 | 7,78% | 467 | 8,77% | 580 | 11,16% | 848 | 10,30% | 793 | 10,66% | 978 |
| OTHER ETHICS ISSUES | 0,96% | 56 | 0,79% | 45 | 1,32% | 79 | 1,07% | 71 | 1,01% | 77 | 1,03% | 79 | 0,95% | 87 |
| DUAL USE | 0,24% | 14 | 0,25% | 14 | 0,10% | 6 | 0,11% | 7 | 0,12% | 9 | 0,10% | 8 | 0,14% | 13 |
| HUMANS | 19,32% | 1124 | 22,76% | 1290 | 24,01% | 1442 | 23,02% | 1522 | 23,06% | 1752 | 24,44% | 1882 | 24,14% | 2215 |
| HUMAN CELLS / TISSUES | 11,07% | 644 | 12,95% | 734 | 13,05% | 784 | 13,17% | 871 | 13,40% | 1018 | 13,09% | 1008 | 12,02% | 1103 |
| ANIMALS | 19,78% | 1151 | 22,27% | 1262 | 21,35% | 1282 | 20,85% | 1379 | 18,14% | 1378 | 16,99% | 1308 | 16,29% | 1494 |
| MISUSE | 0,09% | 5 | 0,14% | 8 | 0,27% | 16 | 0,32% | 21 | 0,51% | 39 | 0,62% | 48 | 0,55% | 50 |
| HUMAN EMBRYOS/FOETUS | 0,48% | 28 | 0,78% | 44 | 0,62% | 37 | 0,54% | 36 | 0,59% | 45 | 0,53% | 41 | 0,47% | 43 |
| CIVIL APPLICATIONS | 0,00% | 0 | 0,00% | 0 | 0,13% | 8 | 0,11% | 7 | 0,16% | 12 | 0,05% | 4 | 0,09% | 8 |

| **Ethics issues self-declared in ITN proposals, by category** | **2014** | | **2015** | | **2016** | | **2017** | | **2018** | | **2019** | | **2020** | |
| --- | --- | --- | --- | --- | --- | --- | --- | --- | --- | --- | --- | --- | --- | --- |
|  | % | # | % | # | % | # | % | # | % | # | % | # | % | # |
| PROTECTION OF PERSONAL DATA | 15,07% | 219 | 15,65% | 308 | 19,34% | 341 | 20,94% | 394 | 20,65% | 395 | 22,69% | 425 | 23,42% | 437 |
| NON-EU COUNTRIES | 24,16% | 351 | 22,26% | 438 | 10,21% | 180 | 7,60% | 143 | 6,38% | 122 | 6,46% | 121 | 7,77% | 145 |
| ENVIRONMENT PROTECTION QUESTION | 6,47% | 94 | 6,45% | 127 | 5,73% | 101 | 6,22% | 117 | 6,22% | 119 | 7,47% | 140 | 7,82% | 146 |
| OTHER ETHICS ISSUES | 0,83% | 12 | 0,71% | 14 | 1,19% | 21 | 0,80% | 15 | 0,52% | 10 | 1,01% | 19 | 0,96% | 18 |
| DUAL USE | 0,48% | 7 | 0,41% | 8 | 0,62% | 11 | 0,32% | 6 | 0,42% | 8 | 0,32% | 6 | 0,27% | 5 |
| HUMANS | 17,34% | 252 | 18,09% | 356 | 22,12% | 390 | 22,64% | 426 | 23,58% | 451 | 22,90% | 429 | 22,62% | 422 |
| HUMAN CELLS / TISSUES | 14,45% | 210 | 15,75% | 310 | 18,15% | 320 | 18,23% | 343 | 18,03% | 345 | 17,35% | 325 | 16,72% | 312 |
| ANIMALS | 20,37% | 296 | 19,72% | 388 | 21,61% | 381 | 21,41% | 403 | 22,43% | 429 | 19,65% | 368 | 18,70% | 349 |
| MISUSE | 0,21% | 3 | 0,10% | 2 | 0,23% | 4 | 0,37% | 7 | 0,47% | 9 | 1,07% | 20 | 0,70% | 13 |
| HUMAN EMBRYOS/FOETUS | 0,62% | 9 | 0,86% | 17 | 0,79% | 14 | 1,33% | 25 | 1,05% | 20 | 0,75% | 14 | 0,96% | 18 |
| CIVIL APPLICATIONS | 0,00% | 0 | 0,00% | 0 | 0,00% | 0 | 0,16% | 3 | 0,26% | 5 | 0,32% | 6 | 0,05% | 1 |

| **Ethics issues self-declared in RISE proposals, by category** | **2014** | | **2015** | | **2016** | | **2017** | | **2018** | | **2019** | | **2020** | |
| --- | --- | --- | --- | --- | --- | --- | --- | --- | --- | --- | --- | --- | --- | --- |
|  | % | # | % | # | % | # | % | # | % | # | % | # | % | # |
| PROTECTION OF PERSONAL DATA | 11,36% | 25 | 10,03% | 40 | 17,29% | 46 | 20,92% | 50 | 21,94% | 43 | 21,05% | 56 | 17,88% | 64 |
| NON-EU COUNTRIES | 50,91% | 112 | 51,38% | 205 | 27,44% | 73 | 24,27% | 58 | 24,49% | 48 | 22,93% | 61 | 25,42% | 91 |
| ENVIRONMENT PROTECTION QUESTION | 9,09% | 20 | 8,27% | 33 | 3,01% | 8 | 5,44% | 13 | 6,12% | 12 | 4,89% | 13 | 8,38% | 30 |
| OTHER ETHICS ISSUES | 0,91% | 2 | 1,00% | 4 | 0,75% | 2 | 1,26% | 3 | 1,02% | 2 | 1,13% | 3 | 0,56% | 2 |
| DUAL USE | 0,45% | 1 | 0,25% | 1 | 0,75% | 2 | 0,00% | 0 | 0,00% | 0 | 0,00% | 0 | 0,00% | 0 |
| HUMANS | 16,36% | 36 | 12,53% | 50 | 22,18% | 59 | 20,08% | 48 | 21,94% | 43 | 22,93% | 61 | 20,95% | 75 |
| HUMAN CELLS / TISSUES | 4,09% | 9 | 8,02% | 32 | 13,53% | 36 | 12,55% | 30 | 11,73% | 23 | 11,28% | 30 | 12,85% | 46 |
| ANIMALS | 6,36% | 14 | 8,52% | 34 | 14,66% | 39 | 15,06% | 36 | 12,24% | 24 | 14,66% | 39 | 13,97% | 50 |
| MISUSE | 0,00% | 0 | 0,00% | 0 | 0,00% | 0 | 0,00% | 0 | 0,51% | 1 | 0,38% | 1 | 0,00% | 0 |
| HUMAN EMBRYOS/FOETUS | 0,45% | 1 | 0,00% | 0 | 0,38% | 1 | 0,00% | 0 | 0,00% | 0 | 0,38% | 1 | 0,00% | 0 |
| CIVIL APPLICATIONS | 0,00% | 0 | 0,00% | 0 | 0,00% | 0 | 0,42% | 1 | 0,00% | 0 | 0,38% | 1 | 0,00% | 0 |
