# Supplementary material for: Ethics appraisal procedure in 79,670 Marie Skłodowska-Curie proposals from the entire European HORIZON 2020 research and innovation program (2014–2020): A retrospective analysis
Source: PLoS One. 2021 Nov 4;16(11):e0259582. doi: 10.1371/journal.pone.0259582 (PMC8568105; doi:10.1371/journal.pone.0259582)
Supplement: S2 File — Data are shown as frequencies and percentages of the total number of proposals with declared ethics issues. (DOCX) [file pone.0259582.s002.docx]

**Supplementary file 2. The comparison between applicants’ awareness of ethics issues as declared in the ethics-self assessment table (“self-declared”) with ethics issues flagged by experts during the ethics review (“after review”) on the main lists, divided per MSCA actions from the entire European HORIZON 2020 research and innovation program (2014 - 2020). Data are shown as frequencies and percentages of the total number of proposals with declared ethics issues**

| **Ethics issues self-declared in COFUND main list, by category** | **2014** | | **2015** | | **2016** | | **2017** | | **2018** | | **2019** | | **2020** | |
| --- | --- | --- | --- | --- | --- | --- | --- | --- | --- | --- | --- | --- | --- | --- |
|  | % | # | % | # | % | # | % | # | % | # | % | # | % | # |
| PROTECTION OF PERSONAL DATA | 18,42% | 7 | 25,71% | 9 | 25,00% | 12 | 16,13% | 5 | 21,74% | 5 | 35,71% | 5 | 29,17% | 14 |
| NON-EU COUNTRIES | 13,16% | 5 | 20,00% | 7 | 10,42% | 5 | 9,68% | 3 | 0,00% | 0 | 7,14% | 1 | 4,17% | 2 |
| ENVIRONMENT PROTECTION QUESTION | 7,89% | 3 | 5,71% | 2 | 6,25% | 3 | 22,58% | 7 | 17,39% | 4 | 14,29% | 2 | 10,42% | 5 |
| OTHER ETHICS ISSUES | 7,89% | 3 | 0,00% | 0 | 4,17% | 2 | 0,00% | 0 | 8,70% | 2 | 7,14% | 1 | 0,00% | 0 |
| DUAL USE | 2,63% | 1 | 0,00% | 0 | 0,00% | 0 | 0,00% | 0 | 0,00% | 0 | 0,00% | 0 | 0,00% | 0 |
| HUMANS | 15,79% | 6 | 14,29% | 5 | 18,75% | 9 | 16,13% | 5 | 13,04% | 3 | 7,14% | 1 | 20,83% | 10 |
| HUMAN CELLS / TISSUES | 10,53% | 4 | 14,29% | 5 | 14,58% | 7 | 9,68% | 3 | 17,39% | 4 | 7,14% | 1 | 14,58% | 7 |
| ANIMALS | 18,42% | 7 | 17,14% | 6 | 18,75% | 9 | 16,13% | 5 | 17,39% | 4 | 21,43% | 3 | 16,67% | 8 |
| MISUSE | 2,63% | 1 | 0,00% | 0 | 0,00% | 0 | 0,00% | 0 | 0,00% | 0 | 0,00% | 0 | 0,00% | 0 |
| HUMAN EMBRYOS/FOETUS | 2,63% | 1 | 2,86% | 1 | 2,08% | 1 | 9,68% | 3 | 4,35% | 1 | 0,00% | 0 | 4,17% | 2 |
| CIVIL APPLICATIONS | 0,00% | 0 | 0,00% | 0 | 0,00% | 0 | 0,00% | 0 | 0,00% | 0 | 0,00% | 0 | 0,00% | 0 |
|  |  |  |  |  |  |  |  |  |  |  |  |  |  |  |
| **Ethics issues after review in COFUND main list, by category** | **2014** | | **2015** | | **2016** | | **2017** | | **2018** | | **2019** | | **2020** | |
|  | % | # | % | # | % | # | % | # | % | # | % | # | % | # |
| PROTECTION OF PERSONAL DATA | 20,85% | 108 | 0,00% | 0 | 0,00% | 0 | 0,00% | 0 | 0,00% | 0 | 0,00% | 0 | 0,00% | 0 |
| NON-EU COUNTRIES | 10,42% | 54 | 0,00% | 0 | 2,94% | 1 | 0,00% | 0 | 0,00% | 0 | 0,00% | 0 | 0,00% | 0 |
| ENVIRONMENT PROTECTION QUESTION | 7,72% | 40 | 0,00% | 0 | 0,00% | 0 | 0,00% | 0 | 0,00% | 0 | 0,00% | 0 | 0,00% | 0 |
| OTHER ETHICS ISSUES | 0,00% | 0 | 3,45% | 1 | 0,00% | 0 | 0,00% | 0 | 0,00% | 0 | 0,00% | 0 | 0,00% | 0 |
| DUAL USE | 1,16% | 6 | 0,00% | 0 | 0,00% | 0 | 0,00% | 0 | 0,00% | 0 | 0,00% | 0 | 0,00% | 0 |
| HUMANS | 20,27% | 105 | 0,00% | 0 | 0,00% | 0 | 0,00% | 0 | 0,00% | 0 | 0,00% | 0 | 0,00% | 0 |
| HUMAN CELLS / TISSUES | 11,39% | 59 | 0,00% | 0 | 0,00% | 0 | 0,00% | 0 | 0,00% | 0 | 0,00% | 0 | 0,00% | 0 |
| ANIMALS | 18,53% | 96 | 0,00% | 0 | 0,00% | 0 | 0,00% | 0 | 0,00% | 0 | 0,00% | 0 | 0,00% | 0 |
| MISUSE | 1,16% | 6 | 0,00% | 0 | 0,00% | 0 | 0,00% | 0 | 0,00% | 0 | 0,00% | 0 | 0,00% | 0 |
| HUMAN EMBRYOS/FOETUS | 8,49% | 44 | 0,00% | 0 | 0,00% | 0 | 0,00% | 0 | 0,00% | 0 | 0,00% | 0 | 0,00% | 0 |
| CIVIL APPLICATIONS | 0,00% | 0 | 0,00% | 0 | 0,00% | 0 | 0,00% | 0 | 0,00% | 0 | 0,00% | 0 | 0,00% | 0 |
| GENERAL | 0,00% | 0 | 96,55% | 28 | 97,06% | 33 | 100,00% | 21 | 100,00% | 29 | 100,00% | 28 | 100,00% | 43 |
|  |  |  |  |  |  |  |  |  |  |  |  |  |  |  |
| **Ethics issues self-declared in IF main list, by category** | **2014** | | **2015** | | **2016** | | **2017** | | **2018** | | **2019** | | **2020** | |
|  | % | # | % | # | % | # | % | # | % | # | % | # | % | # |
| PROTECTION OF PERSONAL DATA | 14,66% | 161 | 17,13% | 147 | 18,34% | 157 | 19,01% | 203 | 17,81% | 202 | 20,34% | 263 | 19,78% | 276 |
| NON-EU COUNTRIES | 22,13% | 243 | 13,75% | 118 | 12,50% | 107 | 12,17% | 130 | 12,35% | 140 | 12,92% | 167 | 13,62% | 190 |
| ENVIRONMENT PROTECTION QUESTION | 8,38% | 92 | 9,67% | 83 | 9,93% | 85 | 9,18% | 98 | 12,26% | 139 | 11,99% | 155 | 13,76% | 192 |
| OTHER ETHICS ISSUES | 0,91% | 10 | 1,05% | 9 | 1,29% | 11 | 0,94% | 10 | 0,88% | 10 | 0,93% | 12 | 1,22% | 17 |
| DUAL USE | 0,18% | 2 | 0,23% | 2 | 0,23% | 2 | 0,09% | 1 | 0,18% | 2 | 0,00% | 0 | 0,22% | 3 |
| HUMANS | 19,67% | 216 | 20,40% | 175 | 23,25% | 199 | 21,44% | 229 | 20,28% | 230 | 22,58% | 292 | 22,44% | 313 |
| HUMAN CELLS / TISSUES | 11,29% | 124 | 13,40% | 115 | 12,03% | 103 | 14,61% | 156 | 14,55% | 165 | 12,68% | 164 | 10,90% | 152 |
| ANIMALS | 22,13% | 243 | 23,43% | 201 | 21,96% | 188 | 21,35% | 228 | 20,28% | 230 | 17,17% | 222 | 17,06% | 238 |
| MISUSE | 0,09% | 1 | 0,12% | 1 | 0,12% | 1 | 0,47% | 5 | 0,35% | 4 | 0,39% | 5 | 0,72% | 10 |
| HUMAN EMBRYOS/FOETUS | 0,55% | 6 | 0,82% | 7 | 0,35% | 3 | 0,66% | 7 | 0,97% | 11 | 0,93% | 12 | 0,22% | 3 |
| CIVIL APPLICATIONS | 0,00% | 0 | 0,00% | 0 | 0,00% | 0 | 0,09% | 1 | 0,09% | 1 | 0,08% | 1 | 0,07% | 1 |
|  |  |  |  |  |  |  |  |  |  |  |  |  |  |  |
| **Ethics issues after review in IF main list, by category** | **2014** | | **2015** | | **2016** | | **2017** | | **2018** | | **2019** | | **2020** | |
|  | % | # | % | # | % | # | % | # | % | # | % | # | % | # |
| PROTECTION OF PERSONAL DATA | 25,99% | 966 | 18,55% | 392 | 24,95% | 482 | 23,45% | 509 | 27,11% | 651 | 27,79% | 764 | 31,42% | 1318 |
| NON-EU COUNTRIES | 14,37% | 534 | 12,26% | 259 | 13,04% | 252 | 12,48% | 271 | 12,20% | 293 | 12,99% | 357 | 10,18% | 427 |
| ENVIRONMENT PROTECTION QUESTION | 6,62% | 246 | 11,50% | 243 | 15,48% | 299 | 19,44% | 422 | 16,70% | 401 | 18,95% | 521 | 20,26% | 850 |
| OTHER ETHICS ISSUES | 2,45% | 91 | 3,64% | 77 | 0,93% | 18 | 1,34% | 29 | 1,37% | 33 | 0,87% | 24 | 0,31% | 13 |
| DUAL USE | 0,56% | 21 | 0,71% | 15 | 0,67% | 13 | 0,28% | 6 | 0,08% | 2 | 0,00% | 0 | 0,19% | 8 |
| HUMANS | 24,80% | 922 | 21,49% | 454 | 23,40% | 452 | 19,16% | 416 | 19,08% | 458 | 21,10% | 580 | 21,79% | 914 |
| HUMAN CELLS / TISSUES | 7,24% | 269 | 7,43% | 157 | 7,25% | 140 | 8,25% | 179 | 9,12% | 219 | 5,97% | 164 | 6,03% | 253 |
| ANIMALS | 16,60% | 617 | 13,63% | 288 | 9,73% | 188 | 10,92% | 237 | 8,70% | 209 | 7,42% | 204 | 7,18% | 301 |
| MISUSE | 0,54% | 20 | 0,57% | 12 | 1,19% | 23 | 1,34% | 29 | 0,54% | 13 | 1,24% | 34 | 0,24% | 10 |
| HUMAN EMBRYOS/FOETUS | 0,83% | 31 | 0,85% | 18 | 0,31% | 6 | 0,51% | 11 | 0,54% | 13 | 0,33% | 9 | 0,05% | 2 |
| CIVIL APPLICATIONS | 0,00% | 0 | 0,00% | 0 | 0,00% | 0 | 0,00% | 0 | 0,00% | 0 | 0,00% | 0 | 0,00% | 0 |
| GENERAL | 0,00% | 0 | 9,37% | 198 | 3,05% | 59 | 2,86% | 62 | 4,54% | 109 | 3,35% | 92 | 2,36% | 99 |
|  |  |  |  |  |  |  |  |  |  |  |  |  |  |  |
| **Ethics issues self-declared in ITN main list, by category** | **2014** | | **2015** | | **2016** | | **2017** | | **2018** | | **2019** | | **2020** | |
|  | % | # | % | # | % | # | % | # | % | # | % | # | % | # |
| PROTECTION OF PERSONAL DATA | 12,42% | 19 | 18,24% | 27 | 16,10% | 19 | 23,35% | 39 | 19,75% | 31 | 21,72% | 43 | 23,24% | 43 |
| NON-EU COUNTRIES | 22,88% | 35 | 16,22% | 24 | 9,32% | 11 | 9,58% | 16 | 3,82% | 6 | 9,09% | 18 | 8,65% | 16 |
| ENVIRONMENT PROTECTION QUESTION | 7,84% | 12 | 8,11% | 12 | 7,63% | 9 | 4,79% | 8 | 7,01% | 11 | 9,60% | 19 | 7,57% | 14 |
| OTHER ETHICS ISSUES | 1,31% | 2 | 0,68% | 1 | 2,54% | 3 | 1,80% | 3 | 0,00% | 0 | 0,51% | 1 | 1,08% | 2 |
| DUAL USE | 1,96% | 3 | 0,68% | 1 | 0,85% | 1 | 0,60% | 1 | 1,27% | 2 | 1,01% | 2 | 0,00% | 0 |
| HUMANS | 14,38% | 22 | 16,89% | 25 | 17,80% | 21 | 25,15% | 42 | 21,66% | 34 | 21,72% | 43 | 23,24% | 43 |
| HUMAN CELLS / TISSUES | 14,38% | 22 | 17,57% | 26 | 18,64% | 22 | 15,57% | 26 | 22,29% | 35 | 17,68% | 35 | 15,68% | 29 |
| ANIMALS | 23,53% | 36 | 21,62% | 32 | 24,58% | 29 | 18,56% | 31 | 22,29% | 35 | 16,67% | 33 | 19,46% | 36 |
| MISUSE | 1,31% | 2 | 0,00% | 0 | 0,85% | 1 | 0,00% | 0 | 0,64% | 1 | 1,52% | 3 | 0,00% | 0 |
| HUMAN EMBRYOS/FOETUS | 0,00% | 0 | 0,00% | 0 | 1,69% | 2 | 0,60% | 1 | 1,27% | 2 | 0,00% | 0 | 1,08% | 2 |
| CIVIL APPLICATIONS | 0,00% | 0 | 0,00% | 0 | 0,00% | 0 | 0,00% | 0 | 0,00% | 0 | 0,51% | 1 | 0,00% | 0 |
|  |  |  |  |  |  |  |  |  |  |  |  |  |  |  |
| **Ethics issues after review in ITN main list, by category** | **2014** | | **2015** | | **2016** | | **2017** | | **2018** | | **2019** | | **2020** | |
|  | % | # | % | # | % | # | % | # | % | # | % | # | % | # |
| PROTECTION OF PERSONAL DATA | 13,26% | 115 | 23,83% | 225 | 16,73% | 47 | 16,62% | 66 | 19,32% | 68 | 14,76% | 58 | 16,63% | 70 |
| NON-EU COUNTRIES | 13,26% | 115 | 10,81% | 102 | 23,84% | 67 | 22,92% | 91 | 12,78% | 45 | 15,52% | 61 | 13,54% | 57 |
| ENVIRONMENT PROTECTION QUESTION | 7,84% | 68 | 9,53% | 90 | 15,66% | 44 | 16,62% | 66 | 17,61% | 62 | 19,59% | 77 | 19,24% | 81 |
| OTHER ETHICS ISSUES | 23,07% | 200 | 3,18% | 30 | 0,71% | 2 | 0,25% | 1 | 0,85% | 3 | 0,25% | 1 | 0,95% | 4 |
| DUAL USE | 1,50% | 13 | 1,06% | 10 | 1,42% | 4 | 1,26% | 5 | 1,14% | 4 | 1,78% | 7 | 0,71% | 3 |
| HUMANS | 16,03% | 139 | 24,05% | 227 | 13,52% | 38 | 15,87% | 63 | 16,19% | 57 | 14,76% | 58 | 14,73% | 62 |
| HUMAN CELLS / TISSUES | 8,42% | 73 | 11,76% | 111 | 11,39% | 32 | 8,82% | 35 | 12,78% | 45 | 10,43% | 41 | 10,21% | 43 |
| ANIMALS | 15,46% | 134 | 14,72% | 139 | 12,46% | 35 | 8,56% | 34 | 11,36% | 40 | 8,91% | 35 | 8,79% | 37 |
| MISUSE | 0,69% | 6 | 0,32% | 3 | 0,71% | 2 | 1,76% | 7 | 1,42% | 5 | 0,25% | 1 | 0,24% | 1 |
| HUMAN EMBRYOS/FOETUS | 0,35% | 3 | 0,74% | 7 | 1,42% | 4 | 0,00% | 0 | 0,57% | 2 | 0,25% | 1 | 0,71% | 3 |
| CIVIL APPLICATIONS | 0,00% | 0 | 0,00% | 0 | 0,00% | 0 | 0,00% | 0 | 0,00% | 0 | 0,00% | 0 | 0,00% | 0 |
| GENERAL | 0,00% | 0 | 0,00% | 0 | 2,14% | 6 | 7,30% | 29 | 5,97% | 21 | 13,49% | 53 | 14,25% | 60 |
|  |  |  |  |  |  |  |  |  |  |  |  |  |  |  |
| **Ethics issues self-declared in RISE main list, by category** | **2014** | | **2015** | | **2016** | | **2017** | | **2018** | | **2019** | | **2020** | |
|  | % | # | % | # | % | # | % | # | % | # | % | # | % | # |
| PROTECTION OF PERSONAL DATA | 14,29% | 15 | 9,60% | 12 | 14,94% | 13 | 13,24% | 9 | 25,93% | 14 | 19,40% | 13 | 14,29% | 10 |
| NON-EU COUNTRIES | 45,71% | 48 | 45,60% | 57 | 25,29% | 22 | 23,53% | 16 | 29,63% | 16 | 26,87% | 18 | 25,71% | 18 |
| ENVIRONMENT PROTECTION QUESTION | 8,57% | 9 | 8,00% | 10 | 1,15% | 1 | 8,82% | 6 | 9,26% | 5 | 4,48% | 3 | 8,57% | 6 |
| OTHER ETHICS ISSUES | 1,90% | 2 | 0,80% | 1 | 1,15% | 1 | 1,47% | 1 | 0,00% | 0 | 1,49% | 1 | 2,86% | 2 |
| DUAL USE | 0,00% | 0 | 0,80% | 1 | 1,15% | 1 | 0,00% | 0 | 0,00% | 0 | 0,00% | 0 | 0,00% | 0 |
| HUMANS | 17,14% | 18 | 12,80% | 16 | 17,24% | 15 | 16,18% | 11 | 22,22% | 12 | 25,37% | 17 | 14,29% | 10 |
| HUMAN CELLS / TISSUES | 4,76% | 5 | 9,60% | 12 | 20,69% | 18 | 14,71% | 10 | 7,41% | 4 | 10,45% | 7 | 15,71% | 11 |
| ANIMALS | 7,62% | 8 | 12,80% | 16 | 17,24% | 15 | 20,59% | 14 | 3,70% | 2 | 11,94% | 8 | 18,57% | 13 |
| MISUSE | 0,00% | 0 | 0,00% | 0 | 0,00% | 0 | 0,00% | 0 | 1,85% | 1 | 0,00% | 0 | 0,00% | 0 |
| HUMAN EMBRYOS/FOETUS | 0,00% | 0 | 0,00% | 0 | 1,15% | 1 | 0,00% | 0 | 0,00% | 0 | 0,00% | 0 | 0,00% | 0 |
| CIVIL APPLICATIONS | 0,00% | 0 | 0,00% | 0 | 0,00% | 0 | 1,47% | 1 | 0,00% | 0 | 0,00% | 0 | 0,00% | 0 |
|  |  |  |  |  |  |  |  |  |  |  |  |  |  |  |
| **Ethics issues after review in RISE main list, by category** | **2014** | | **2015** | | **2016** | | **2017** | | **2018** | | **2019** | | **2020** | |
|  | % | # | % | # | % | # | % | # | % | # | % | # | % | # |
| PROTECTION OF PERSONAL DATA | 22,12% | 50 | 20,57% | 136 | 23,62% | 146 | 27,10% | 116 | 38,28% | 209 | 29,94% | 156 | 26,23% | 96 |
| NON-EU COUNTRIES | 26,99% | 61 | 29,65% | 196 | 18,77% | 116 | 20,79% | 89 | 15,75% | 86 | 19,77% | 103 | 23,77% | 87 |
| ENVIRONMENT PROTECTION QUESTION | 4,42% | 10 | 8,62% | 57 | 8,09% | 50 | 9,35% | 40 | 7,69% | 42 | 8,25% | 43 | 12,30% | 45 |
| OTHER ETHICS ISSUES | 7,08% | 16 | 2,27% | 15 | 0,32% | 2 | 0,47% | 2 | 0,73% | 4 | 0,19% | 1 | 1,37% | 5 |
| DUAL USE | 0,00% | 0 | 3,78% | 25 | 1,29% | 8 | 0,00% | 0 | 0,00% | 0 | 0,96% | 5 | 2,19% | 8 |
| HUMANS | 22,12% | 50 | 19,06% | 126 | 23,46% | 145 | 25,00% | 107 | 26,19% | 143 | 22,07% | 115 | 19,40% | 71 |
| HUMAN CELLS / TISSUES | 6,64% | 15 | 6,96% | 46 | 12,94% | 80 | 6,07% | 26 | 5,31% | 29 | 10,56% | 55 | 5,46% | 20 |
| ANIMALS | 10,62% | 24 | 7,72% | 51 | 8,09% | 50 | 6,78% | 29 | 1,47% | 8 | 4,22% | 22 | 7,38% | 27 |
| MISUSE | 0,00% | 0 | 1,06% | 7 | 0,49% | 3 | 0,23% | 1 | 1,47% | 8 | 0,19% | 1 | 0,82% | 3 |
| HUMAN EMBRYOS/FOETUS | 0,00% | 0 | 0,00% | 0 | 0,16% | 1 | 0,00% | 0 | 0,00% | 0 | 0,00% | 0 | 0,00% | 0 |
| CIVIL APPLICATIONS | 0,00% | 0 | 0,00% | 0 | 0,00% | 0 | 0,00% | 0 | 0,00% | 0 | 0,00% | 0 | 0,00% | 0 |
| GENERAL | 0,00% | 0 | 0,15% | 1 | 2,75% | 17 | 4,21% | 18 | 3,11% | 17 | 3,84% | 20 | 1,09% | 4 |
