# Supplementary material for: Ethics appraisal procedure in 79,670 Marie Skłodowska-Curie proposals from the entire European HORIZON 2020 research and innovation program (2014–2020): A retrospective analysis
Source: PLoS One. 2021 Nov 4;16(11):e0259582. doi: 10.1371/journal.pone.0259582 (PMC8568105; doi:10.1371/journal.pone.0259582)
Supplement: S3 File — (DOCX) [file pone.0259582.s003.docx]

**Supplementary file 3. Detailed explanation of each outcome category as shown in Table 3 and Table 4**

| Inadmissible/ineligible | *see explanation in “methods”* |
| --- | --- |
| No self-declared ethics issues, no screening because of low ranking | *applicant did not provide any self-declaration, the proposal was not screened by experts for ethics issues as the ranking was too low (below available budget or below 70% threshold)* |
| Self-declared ethics issues, no screening because of low ranking | *applicant provided one or more self-declarations, the proposal was not screened by experts for ethics issues as the ranking was too low (below available budget or below 70% threshold)* |
| Proposals screened | *all proposals that went through the screening process* |
| No self-declared ethics issues, incomplete data | *applicant did not provide any self-declaration, the database could not extract complete data on requirements* |
| No self-declared ethics issues, screening resulted in "ethics clearance" | *applicant did not provide any self-declaration, ethics experts screened the proposal and found it did not need additional requirements, so it was cleared* |
| No self-declared ethics issues, screening resulted in "conditional clearance" with requirements | *applicant did not provide any self-declaration, ethics experts screened the proposal and found it needed additional requirements, to be submitted as project deliverables* |
| No self-declared ethics issues, screening resulted in "conditional clearance" with requirements, ethics check added | *applicant did not provide any self-declaration, ethics experts screened the proposal and found it needed additional requirements, to be submitted as project deliverables, and in addition suggested an ethics check be performed during the project lifetime* |
| Self-declared ethics issues, incomplete data | *applicant provided one or more self-declarations, the database could not extract complete data on requirements* |
| Self-declared ethics issues, screening resulted in "ethics clearance" | *applicant provided one or more self-declarations, ethics experts screened the proposal and found it did not need additional requirements, so it was cleared* |
| Self-declared ethics issues, screening resulted in "conditional clearance" with requirements | *applicant provided one or more self-declarations, ethics experts screened the proposal and found it needed additional requirements, to be submitted as project deliverables* |
| Self-declared ethics issues, incomplete data, screening resulted in "conditional clearance" with requirements, ethics check added | *applicant provided one or more self-declarations, ethics experts screened the proposal and found it needed additional requirements, to be submitted as project deliverables, and in addition suggested an ethics check be performed during the project lifetime* |
| Self-declared ethics issues, screening resulted in "conditional clearance" with requirements, ethics check added | *applicant provided one or more self-declarations, ethics experts screened the proposal and found it needed additional requirements, to be submitted as project deliverables, and in addition suggested an ethics check be performed during the project lifetime* |
| Self-declared ethics issues, screening resulted in "no clearance" | *applicant provided one or more self-declarations, ethics experts screened the proposal and found it could not receive ethics clearance and therefore could not be funded* |
