# Supplementary material for: Ethics appraisal procedure in 79,670 Marie Skłodowska-Curie proposals from the entire European HORIZON 2020 research and innovation program (2014–2020): A retrospective analysis
Source: PLoS One. 2021 Nov 4;16(11):e0259582. doi: 10.1371/journal.pone.0259582 (PMC8568105; doi:10.1371/journal.pone.0259582)
Supplement: Attachment — Submitted filename: 2021_09_14 Response 2.docx [file pone.0259582.s004.docx]

Roland Paile Bendaña
PLOS ONE

**Re: Revision of the manuscript PONE-D-21-15555R1**

Dear Editor,

As requested, we made additional revisions:

1. Ethics statement is now only in the Methods section of the manuscript.

2. We revised Data availability statement, and we provided response to the four questions.

Sincerely,

The authors

PONE-D-21-15555R1
Ethics appraisal procedure in 79,670 Marie Skłodowska-Curie proposals from the entire European HORIZON 2020 research and innovation program (2014 - 2020): a retrospective analysis
Prof.Dr. Zvonimir Koporc

Dear Dr. Koporc,

We've checked your submission and before we can proceed, we need you to address the following issues:

1. Your ethics statement should only appear in the Methods section of your manuscript. If your ethics statement is written in any section besides the Methods, please delete it from any other section.

2. We note your current Data Availability Statement: “Raw data used in this study belong to European Union's Research Executive Agency (REA). The REA approved use of the data solely for the purpose of this study, and not for public sharing. Among the four study authors, there are two members of REA (IDW, DW) and they had access to raw data at all times. The remaining two authors (LP, ZK) do not have raw data to share. For the purpose of the study, only anonymized summary data were presented. For access to raw anonymized data (i.e. for validation purposes or for new research projects), additional approval from the REA is required. Researchers interested in these data are invited to contact the corresponding author for further information about access to the data set."

Thank you for further specifying the restrictions placed on your data. For studies involving third-party data, we encourage authors to share any data specific to their analyses that they can legally distribute. PLOS recognizes, however, that authors may be using third-party data they do not have the rights to share. When third-party data cannot be publicly shared, authors must provide all information necessary for interested researchers to apply to gain access to the data. (<https://journals.plos.org/plosone/s/data-availability#loc-acceptable-data-access-restrictions>)

For any third-party data that the authors cannot legally distribute, they should include the following information in their Data Availability Statement upon submission:
1. A description of the data set and the third-party source
2. If applicable, verification of permission to use the data set
3. Confirmation of whether the authors received any special privileges in accessing the data that other researchers would not have
4. Direct, non-author contact information through which interested researchers can request data access. Please note that in the interest of long-term data availability, PLOS data policy states that it is not acceptable for an author to be the sole named individual responsible for ensuring data access.

Kind regards,
Roland Paile Bendaña
PLOS ONE
